# The liver metastatic niche: modelling the extracellular matrix in metastasis

**DOI:** 10.1242/dmm.048801

**Published:** 2021-04-15

**Authors:** James Drew, Laura M. Machesky

**Affiliations:** 1 CRUK Beatson Institute, Switchback Road, Bearsden, Glasgow G61 1BD, UK; 2 Institute of Cancer Sciences, University of Glasgow, Glasgow G61 1QH, UK

**Keywords:** Cancer metastasis, Extracellular matrix, Liver metastasis, Metastatic niche

## Abstract

Dissemination of malignant cells from primary tumours to metastatic sites is a key step in cancer progression. Disseminated tumour cells preferentially settle in specific target organs, and the success of such metastases depends on dynamic interactions between cancer cells and the microenvironments they encounter at secondary sites. Two emerging concepts concerning the biology of metastasis are that organ-specific microenvironments influence the fate of disseminated cancer cells, and that cancer cell-extracellular matrix interactions have important roles at all stages of the metastatic cascade. The extracellular matrix is the complex and dynamic non-cellular component of tissues that provides a physical scaffold and conveys essential adhesive and paracrine signals for a tissue's function. Here, we focus on how extracellular matrix dynamics contribute to liver metastases – a common and deadly event. We discuss how matrix components of the healthy and premetastatic liver support early seeding of disseminated cancer cells, and how the matrix derived from both cancer and liver contributes to the changes in niche composition as metastasis progresses. We also highlight the technical developments that are providing new insights into the stochastic, dynamic and multifaceted roles of the liver extracellular matrix in permitting and sustaining metastasis. An understanding of the contribution of the extracellular matrix to different stages of metastasis may well pave the way to targeted and effective therapies against metastatic disease.

## Abstract (precis)

**Summary:** Extracellular matrix dynamics are emerging as a key feature of metastasis. We discuss the changes in matrix composition of the liver metastatic niche, how these impact disease progression, and the approaches to study this topic.

## Introduction

Metastatic disease is the most common cause of death for most solid tumour patients, and highly metastatic cancers such as pancreatic adenocarcinoma (PDAC) and colorectal cancer (CRC) have particularly poor 5-year survival rates that worsen with metastatic burden (Dillekås et al., 2019). Although therapies specifically targeting metastatic disease are still uncommon, our understanding of how cancer cells escape the primary tumour and establish themselves in secondary sites has improved significantly over the past two decades. Metastasis of solid tumours requires intravasation of tumour cells into blood or lymphatic vessels, survival in circulation and subsequent extravasation, followed by dormancy (see Glossary, Box 1) or growth at distant sites.

**Box 1. Glossary**

**Basement membrane (BM):** the extracellular matrix (ECM) structure produced by epithelial cells, endothelial cells, adipocytes and other cell types, which provides structure to a tissue and maintains the polarity and identity of adjacent cells.

**Dormancy:** a net non-proliferative state in which cancer cells can survive immune clearance and chemotherapies, and can reawaken to initiate metastases in response to external cues.

**Epithelial-to-mesenchymal transition (EMT):** also referred to as epithelial-to-mesenchymal plasticity. The transition of cells between epithelial and mesenchymal cell states in which cells lose their polarity and gain an increased invasive capacity. This transition defines broad transcriptional profiles and changes in cell-cell contacts, migration and proliferation. The same cellular processes are also involved in the reverse, mesenchymal-to-epithelial transition.

**Genetically engineered mouse models (GEMMs):** mouse models with genetic lesions that predispose them to tumourigenesis. Genetic lesions can be either be global (e.g. *Tp53* knockout) or targeted to a particular tissue or cell type (e.g. Pdx1-Cre in the KPC mouse model of pancreatic cancer).

**Hepatic stellate cell (HSC):** a mesenchymal cell type occupying the space of Disse in the liver sinusoids. During inflammation, fibrosis or metastasis, these cells can transdifferentiate to activated myofibroblasts, leading to bulk secretion of extracellular matrix.

**Hepatocyte:** the major parenchymal cell type of the liver that performs the majority of metabolic functions.

**Interstitial ECM:** ECM structures that support the bulk tissue of organs. This matrix is primarily produced by the parenchymal cells, i.e. hepatocytes in the liver.

**Kupffer cell:** liver-resident macrophage lining the sinusoidal vessels.

**Liver metastatic niche (LMN):** the combined cellular and acellular microenvironment occupied by tumour cells that have disseminated to the liver. The niche is not static, but changes in response to cues from cancer cells and external factors (e.g. infiltrating immune cells).

**Matrisome:** the collection of >1000 genes that produce the ECM components or the proteins that directly interact with them. The matrisome was collated through proteomic analyses of decellularised tissues.

**Microphysiological system (MPS):** *in vitro* models that aim to recapitulate the key features of *in vivo* tissues, including 3D architecture, cellular composition and blood flow.

**Organoids and spheroids:** self-organised 3D cell cultures generated from stem cells or primary tumour cells. Organoids typically contain multiple cell types and recapitulate the tissue architecture of an organ, whereas spheroids are cellular aggregates typically composed of a single type of differentiated but immortalised cells, such as cancer cells or cell lines.

**Sinusoid:** sinusoids are low-pressure fenestrated capillaries in the liver that link the hepatic artery and portal vein at the periphery of lobules to deliver blood into central veins. Sinusoids are lined with endothelial cells and flanked by hepatocytes.

**Space of Disse:** area between the sinusoid and hepatocytes in the liver. In healthy liver, the space is occupied by hepatic stellate cells and microvilli from hepatocytes.

**Transplant model:** models in which malignant cell lines or primary cells are transplanted into animals, most often mice. Orthotopic models generate primary tumours, whereas injecting cells into the circulation, e.g. into the tail vein or spleen, provides rapid-onset metastases in specific target organs.

Disseminated tumour cells (DTCs) preferentially settle in specific organs driven by factors including tissue mechanical properties (Reuten et al., 2021), vascular physiology (Follain et al., 2018) and microenvironment (Ingangi et al., 2019). Conceptualised in Paget's ‘soil and seed’ hypothesis, there is now ample evidence that progression of metastases requires favourable local conditions in different organs that develop to form the ‘metastatic niche’ (Paget, 1889). Our current understanding of this phenomenon is largely based on the lung metastatic niche and metastatic breast cancer. These studies have highlighted the importance of niche-specific cell types and tissue structures in metastatic progression (Montagner et al., 2020; Altorki et al., 2019). However, the liver is another metastatic site that warrants study owing to, first, its involvement in aggressive cancers including pancreatic, colorectal, breast and lung cancer; second, its association with poor survival compared with other metastatic sites; and third, the liver's unique architecture, vascular physiology and cell composition (Budczies et al., 2015; Sahin et al., 2018). Understanding how features of the liver metastatic niche (LMN; Box 1) help support metastases from tumours of different primary origin is of vital importance in the development of effective therapies against metastatic disease.

A key component of the metastatic niche is the extracellular matrix (ECM) – the collection of extracellular proteins that provide the three-dimensional (3D) scaffold within which cells organise to form complex structures. Historically, ECM has referred to the families of fibrillar proteins (collagens), glycoproteins (fibronectin, laminins) and proteoglycans (heparin sulphate proteoglycans, versican) that form a dynamic interface for mechanical and biochemical interactions with cells. This view has recently expanded to include proteins that regulate ECM secretion, processing, remodelling, degradation and binding – collectively referred to as the matrisome (Box 1) (Naba et al., 2012). Indeed, remodelling of the ECM can release growth factors and signalling molecules trapped within it, linking ECM dynamics to a plethora of biological consequences for the cells.

DTCs utilise ECM dynamics to create a supportive niche within which they can survive, evade immune destruction and eventually proliferate. To date, most studies of the ECM in liver metastasis have focused either on the most abundant fibrillar ECM components or on the broader effects of ECM deposition on tissue mechanics (Shen et al., 2020), immune penetration (Hu et al., 2019) and metastatic burden. What is less clear is how ECM dynamics develop as metastasis progresses and which cell-ECM interactions are particularly relevant. Studies comparing the cellular origins of ECM in primary tumours and metastatic niches have revealed contributions from cancer cells (Tian et al., 2020), fibroblasts (Costa-Silva et al., 2015) and other non-mesenchymal cells of the tumour microenvironment (Hoshino et al., 2015). This is supported by reports of unique ECM profiles of liver metastases from tumours of different origins (Yuzhalin et al., 2019; Hebert et al., 2020; Tian et al., 2019). Indeed, despite sharing clonal origins within primary tumours, protein abundance signatures from liver metastases are unique and favour cellular programmes involved in ECM-receptor interactions and cell adhesion (Li et al., 2020).

We propose that several key questions need to be addressed to better understand the liver metastatic niche. (1) What is the functional relevance of cell type-specific matrisome secretion? (2) How does the ECM compartment of the LMN develop during metastatic progression? (3) Do any cell-ECM interactions represent bottlenecks or decision points in the metastatic fate? Tackling these questions will advance our understanding of the roles of the ECM in the metastatic process and hopefully identify novel therapeutic targets for anti-metastatic treatments.

This Review focuses on how ECM dynamics in the liver metastatic niche emerge and how these changes determine metastatic fate. Of note, we do not aim to cover the extensive literature showing the general impact of matrix stiffness, integrin-adhesion complexes and specific ECM-cell interactions on various aspects of cancer cell biology, which have been extensively reviewed elsewhere (Leight et al., 2017; Nallanthighal et al., 2019; Zanconato et al., 2019; Papalazarou et al., 2018). Instead, we highlight the different models and technical approaches used to study these processes, and hope that this discussion provides a basis for a niche-centric perspective on ECM dynamics during metastasis.

## Systems for studying metastatic ECM

A number of different model systems are now available to researchers interested in ECM dynamics and liver metastasis (Table 1). These fall on a spectrum that balances physiological relevance with ease of use and adaptability. Until recently, the workhorse of cancer biology research has arguably been 2D cell cultures derived from tumours, which, although useful for studying fundamental cancer cell biology, do not accurately recapitulate cell-ECM interactions in tumours.

**Table 1.**
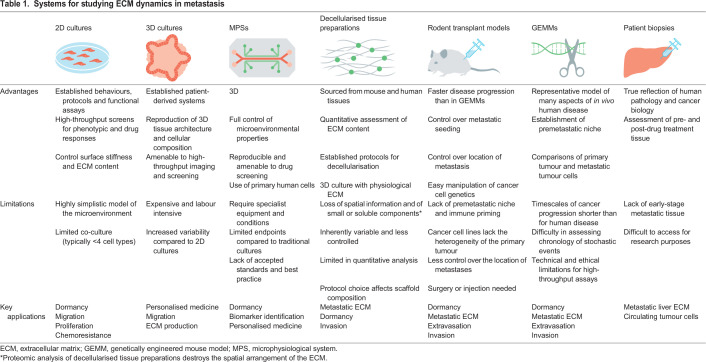
**Systems for studying ECM dynamics in metastasis**

Three-dimensional cell cultures can better model the physical properties of tissues and the heterogeneity of the cells and ECM within them. Organoids (Box 1) partially re-create the microstructure of organs, including the liver (Hu et al., 2019). Their cellularity is limited by the potency of the stem cell population and usually they require embedding in an exogenous ECM substrate, e.g. the commercially available Matrigel. Spheroids (Box 1) can contain several predefined cell types at the expense of a developmentally programmed microstructure (Lucendo-Villarin et al., 2019). The inclusion of immune cells in both organoid (Neal et al., 2018) and spheroid (Yin et al., 2016) systems can further recapitulate the tumour microenvironment. Both malignant organoids and spheroids secrete more ECM than 2D cultures and form microenvironments with similarities to their *in vivo* counterparts (Longati et al., 2013).

Microphysiological systems (MPSs; Box 1), also known as organ-on-a-chip, rely on advances in 3D printing and small-scale bioengineering to further model tissue architecture and multi-organ systems. In particular, MPSs excel at modelling structural tissue properties such as blood flow and nutrient gradients, which have been shown to regulate DTC-endothelial cell interactions during extravasation (Follain et al., 2018; Ando et al., 2017). Although significant efforts have been made to develop liver-on-a-chip models (Jang et al., 2019; Gori et al., 2016), to date there are few examples of their applications in metastasis research.

Despite these advances, tissue-based systems remain the gold standard for studying the composition and structure of metastatic ECM. Patient biopsies (Naba et al., 2014), genetically engineered mouse models (GEMMs; Box 1) (Wishart et al., 2020) and cancer cell transplant models (Box 1) (Hebert et al., 2020) all generate metastatic tissue samples that can be used to characterise ECM state. An important recent advance is decellularisation – removing cells from these tissues without compromising the extracellular compartment. Decellularised tissues enable quantitative profiling of the matrisome (Naba et al., 2017) and multiplexed imaging of the 3D organisation of the ECM (Mayorca-Guiliani et al., 2019), and can be used as a 3D culture scaffold to reintroduce cells into (Wishart et al., 2020). Although they are informative, there are limitations to their use. Current proteomic applications are limited by the need for gross changes in ECM to detect differences compared to control tissues, precluding their use for characterising localised changes, such as those that might occur early in metastasis. Decellularised tissues also represent a static snapshot of the ECM state and are of limited use in assessing ECM dynamics. Finally, retention of ECM proteins is protocol and tissue dependent (Krasny et al., 2016), which can affect experimental results.

The stochastic nature of metastasis has made tracking its progression within tissue samples challenging. Thus, the majority of research has relied on bulk metastatic burden as a primary endpoint. Although this research has been informative, it has led to a relatively poor understanding of the early events within the metastatic niche. Functional insight into genes associated with liver metastasis has also been limited and has mainly relied on analyses of tissue transcriptomics and patient survival. How the different models described here have begun to expand our knowledge of the metastatic cascade is discussed below.

## Profiling the liver matrisome during metastasis

### Healthy liver structure and ECM

**Fig. 1.**
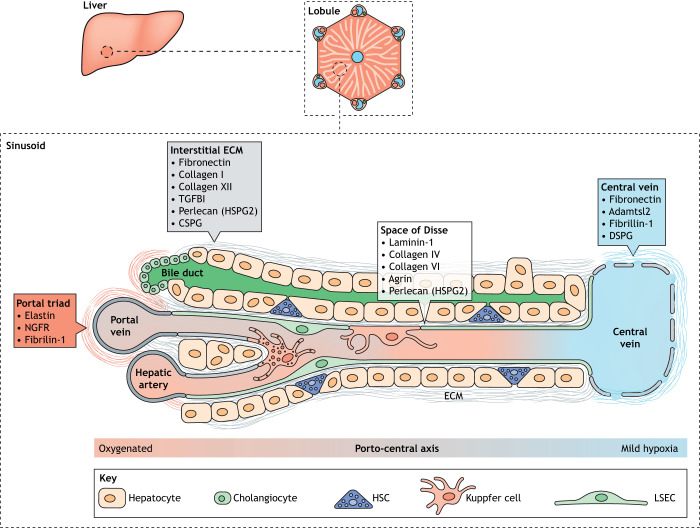
**Liver structure, and cellular and common ECM proteins.** Liver tissue is organised into discrete functional units called lobules that have a hexagonal arrangement of portal triads around a central vein, linked by sinusoids. LSECs line the sinusoid, whereas HSCs occupy the space of Disse that separates the sinusoid from hepatocytes. The different microstructures within the sinusoid have unique ECM compositions, shown in the boxes. A partial basement membrane lines the space of Disse, facilitating exchange of nutrients, proteins and xenobiotics. The interstitial matrix includes typical ECM components such as fibronectin and collagen I that support tissue structure and integrity. Differences also exist in ECM composition along the centroportal axis, although the functional relevance of this is unclear. CSPG, chondroitin sulfate proteoglycan 4; DSPG, dermatan sulfate proteoglycan; ECM, extracellular matrix; HSC, hepatic stellate cell; HSPG2, heparan sulfate proteoglycan core protein 2; LSEC, liver sinusoid endothelial cell; NGFR, tumour necrosis factor receptor superfamily member 16; TGFB1, transforming growth factor beta 1. The sinusoid and lobule structures are adapted from Frevert et al. (2005) and the Wikipedia Commons file 201904_hepatic_lobule.svg under the terms of the CC-BY 2.5 and 4.0 license, respectively.

The liver is the largest internal organ in the body and the major site of drug metabolism, blood detoxification, glucose storage and bile production (Stanger, 2015). These functions are facilitated by a hierarchical, 3D structure of liver lobes, lobules and lobule substructures (Fig. 1). Blood enters the organ via two vessels: the hepatic artery, which delivers fresh blood, and the portal vein, which delivers blood from the gastrointestinal system, gallbladder, pancreas and spleen. These major vascular systems converge in a network of sinusoids (Box 1). Sinusoids have a polar arrangement, with the portal triad (hepatic artery, portal vein, bile duct) at one end and a central vein at the other, which takes blood away from the liver. Hepatocytes (Box 1) are the major epithelial parenchymal cell in the liver, constituting ∼60% of all cells, and fill the space in-between the sinusoids. Another epithelial cell population called cholangiocytes line the bile duct. Additionally, the liver contains various non-parenchymal cells that support parenchymal cell function, including liver sinusoidal endothelial cells (LSECs), a resident macrophage population called Kupffer cells (Box 1) and hepatic stellate cells (HSCs; Box 1), a type of mesenchymal fibroblast-like cell, which occupy the space of Disse (Box 1). An important consequence of liver lobule polarisation is the hypoxic gradient that exists from the highly oxygenated cells near the portal triad to the mildly hypoxic ones near the central vein (Fig. 1). This zonation has well-established consequences for the metabolic functions of hepatocytes; periportal hepatocytes conduct oxygen-intensive functions such as glycogen metabolic and amino acid utilisation, whereas pericentral hepatocytes perform glycolysis and glutamine synthesis (Jungermann and Katz, 1989). Recently, the position along the portocentral axis has been shown to also affect gene expression in LSECs (Aizarani et al., 2019) and HSCs (Dobie et al., 2019). Whether this heterogeneity influences interactions with DTCs is currently unknown.

The healthy liver is thought to have a relatively minimal ECM component. The sinusoidal endothelium lacks a typical complete basement membrane (BM; Box 1) to facilitate material exchange, although the key fibrillar ECM proteins such as laminin, collagen IV and fibronectin line the sinusoids (Pozzi et al., 2017; Rosenow et al., 2008). Recent proteomic studies of the heathy liver matrisome have revealed a more diverse set of core matrisome components including collagens, fibulins, annexins and elastins (Goddard et al., 2016; Krasny et al., 2016; Naba et al., 2014). Collagens I, IV and VI are particularly abundant, with collagens IV and VI forming part of the liver's BM structures, whereas collagen I is found more predominantly in the interstitial ECM (Box 1). The organisation of interstitial collagens has recently been imaged in 3D using decellularised liver preparations (Mayorca-Guiliani et al., 2019), revealing that collagens I, VII and XIV have unique patterns characterised by different levels of fibre bundling and alignment that determine their effects on tissue mechanics. It is worth noting that these ECM factors are secreted by hepatocytes and cholangiocytes, not HSCs, in the healthy liver. Single-cell RNA sequencing (RNAseq) of murine HSCs has, however, identified a number of matrisome components differentially expressed along the centroportal axis; *Podn*, *Loxl1* and *Adamtsl2* show pericentral expression, whereas *Igfbp3* and *Itgb3* are periportal (Dobie et al., 2019). The functional relevance of these asymmetries is currently unknown, but warrants further study.

Liver fibrosis is one of the most extensively studied examples of aberrant ECM accumulation, and has been suggested as a model for understanding ECM changes in liver metastasis (Lee et al., 2019; Zhao et al., 2017). A detailed discussion of ECM changes in liver fibrosis is beyond the scope of this Review and has been excellently described recently (Arteel and Naba, 2020). However, several developments in the field have direct relevance to the study of metastatic ECM changes in the liver. First, proteomic analyses of liver tissue at different stages of fibrosis has shown that different ECM proteins are enriched at specific times in the fibrotic cascade (Baiocchini et al., 2016). Second, although HSCs are the major depositors of ECM during liver fibrosis, recent studies have identified spatially distinct subpopulations of HSCs that respond to chemically induced fibrosis (Mederacke et al., 2013; Dobie et al., 2019). The activation of HSCs is partly driven by inflammatory signals from resident and invading immune cells (Tsuchida and Friedman, 2017). Finally, there is extensive evidence for reversal of severe liver fibrosis in humans and preclinical models (Iredale et al., 1998; Schiff et al., 2011). The signalling pathways underlying such reversal could have particular relevance in targeting ECM deposition during metastasis. Reversal of fibrosis also points to the adaptability and dynamism of the liver ECM, even in disease states. Combined, these studies highlight the importance of understanding spatial and temporal aspects of ECM dynamics in the liver.

### The liver premetastatic niche

The concept that cancers can influence the biology of distant organs through their shared circulation even before cells disseminate from the primary tumour is an emerging and sometimes controversial area of study. Communication from the primary tumour has been proposed to create a more favourable environment for metastatic seeding, known as the premetastatic niche (PMN). The signals that generate PMNs have organ-specific features, but converge on common events such as infiltration of bone marrow-derived and immune cells, and ECM changes (Peinado et al., 2017).

As PMN formation requires both an intact immune system and a primary tumour, genetically engineered mice and orthotopic injection models have been critical to researching this phenomenon. The KPC mouse is a pancreatic cancer model in which targeted mutations in *Tp53* (also known as *Trp53*) and *Kras* lead to PDAC and liver metastases (Hingorani et al., 2005). This model was used recently to show that livers of tumour-bearing mice have enhanced fibronectin and collagen I deposition prior to overt metastases (Lee et al., 2019). RNAseq of these livers found altered expression of transcripts encoding other matrisome components, including matrix metalloproteinase 9 (Mmp9) and S100a proteins (Lee et al., 2019), which were previously co-associated with metastasis at other sites (Lloyd et al., 1998; Zhang et al., 2011). These changes depend on an interleukin-6 (IL-6)/phospho-STAT3/serum amyloid A (SAA) signalling cascade in hepatocytes (Lee et al., 2019), which has also been implicated in CRC liver metastases (Lin et al., 2019). Interestingly, this signalling pathway has well-established roles in liver regeneration (Klein et al., 2005) and innate immunity (Zhou et al., 2016). Indeed, although the origin of premetastatic ECM deposition is currently unclear, IL-6 and SAA proteins are both involved in HSC-mediated fibrosis during the acute-phase response, which is the early inflammatory response to injury or infection (Siegmund et al., 2016; Xiang et al., 2018). Thus, signals from the primary tumour appear to hijack innate immune and homeostatic pathways in hepatocytes to drive ECM changes prior to the arrival of DTCs.

Another emerging concept is that fibronectin deposition in the liver PMN could be caused by signalling from extracellular vesicles (EVs) secreted by the primary tumour. Pre-treating mice with PDAC-derived EVs enhanced subsequent seeding of PDAC DTCs in the liver in an intrasplenic injection model (Costa-Silva et al., 2015). The authors showed that EV-derived macrophage migration inhibitory factor is taken up by Kupffer cells to initiate a fibrotic cascade driven by transforming growth factor β (TGF-β) signalling to HSCs. Indeed, the abundance of integrin αvβ5 in EVs directs uptake in the liver over lung or brain through its interaction with Kupffer cells (Hoshino et al., 2015). Additionally, EVs in the duct fluid of PDAC patients have also been shown to contain ECM components, including tenascin-C, MMP7 and laminins, pointing to a direct contribution to ECM from primary tumours (Zheng et al., 2018).

ECM changes in the liver PMN appear to be critical for immune cell recruitment. Increased fibronectin deposition promotes macrophage infiltration into the liver, a precursor step to PMN formation (Costa-Silva et al., 2015). Additionally, the metalloprotease inhibitor TIMP1, released by primary CRC tumours, induces neutrophil recruitment via a stromal cell-derived factor 1/C-X-C chemokine receptor 4 signalling axis in the liver prior to cancer cell arrival (Grünwald et al., 2016; Seubert et al., 2015). As well as promoting myofibroblast transformation of HSCs, macrophages and neutrophils also secrete a unique combination of matrisome proteins that facilitate migration via ECM degradation (Etich et al., 2019; Nielsen et al., 2016). The relevance of direct ECM deposition by immune cells is currently unclear, but could significantly contribute to local ECM remodelling given the abundance of these cells in the PMN.

Thus, altered ECM is a canonical feature of the liver PMN that is required for its pro-metastatic effects. Our understanding of these changes remains very rudimentary, and a comprehensive assessment of the matrisome signature of the liver PMN is still lacking. In humans, liver metastases can often occur on a backdrop of liver disease-mediated fibrosis. Whether this causally promotes metastasis remains unresolved, but understanding the relationship between fibrosis in liver disease versus pre-metastatic livers is a fruitful avenue for future research (Kondo et al., 2016).

### The metastatic liver ECM

Metastatic disease dramatically alters the cellular and extracellular composition of the liver. Indeed, unbiased transcriptomics and proteomics of human CRC liver metastases and primary tumours have identified ECM and cell adhesion programmes as the key differentially expressed pathways (Lin et al., 2011; Del Rio et al., 2013; Li et al., 2020).

These changes have recently been elucidated in spectacular detail through advances in decellularisation techniques, enabling proteomic analyses of the decellularised ECM (dECM) of healthy and metastatic livers. One such study using CRC patient tissue identified 56 matrisome proteins that were upregulated in metastatic livers, several of which were not expressed in either primary tumour or normal liver samples (Naba et al., 2014). These included both core matrisome (Comp, Fndc1, Igfals, Spp1) and matrisome-associated (Bmp1, C1qtnf5, Hpx) proteins, and suggest that unique ECM profiles can arise from the interactions between DTCs and the liver stroma. A similar study using intrasplenic injection of the CRC cell line MC38 identified a 13-protein ECM signature of liver metastases that included S100a proteins 4, 6 and 11, annexins 1 and 2, and a number of glycoproteins, such as Thbs3, Sparc, Emilin2 and Fbln2 (Yuzhalin et al., 2019).

DTCs of different origins produce unique matrisome signatures distinct from other cancer types and from the normal liver stroma. Orthotopic or injection models of human cancer cell lines into severe combined immunodeficient (SCID) or nude mice allows the separation of matrisome signatures from the host stromal ECM through identification of species-specific peptides. This approach was recently used to show that the liver, lung, brain and bone metastases disseminated upon injection of the human breast cancer cell line MDA-MB-231 had markedly different ECM signatures compared to both the original cancer cells and the host stroma (Hebert et al., 2020). This elegantly shows the importance of the metastatic niche in influencing the ECM identity of cancer cells. The liver metastasis-specific ECM signature included cancer cell-derived collagen VI and stroma-derived tenascin-C, collagen VI and collagen XIV, fibronectin and fibrinogens. Interestingly, collagen VI is one of the more abundant ECM components of normal liver and has been associated with metastasis in breast cancer (Wishart et al., 2020) and PDAC (Owusu-Ansah et al., 2019) but the functional relevance of these liver-specific ECM components remains unclear. However, knockdown of the brain metastasis-enriched SERPINB1 specifically reduced brain metastases (Hebert et al., 2020), implying that these unique signatures do have functional relevance.

Growth patterns of liver metastases also differ greatly depending on their origin. Liver metastases from breast cancers typically adopt a ‘replacement’ strategy whereby cancer cells take over the space occupied by hepatocytes and co-opt the existing sinusoid vasculature (Stessels et al., 2004). In contrast, CRC liver metastases are more disruptive of the overall liver architecture, with a subset of these CRC metastases developing distinct ‘desmoplasmic’ accumulations of ECM between the invasive front and the liver stroma, which is also seen in PDAC liver metastases (Vermeulen et al., 2001; Whatcott et al., 2015). Although these histopathological subtypes have distinct ECM distributions, a systematic comparison of matrisome compositions is lacking and may shed light on why different growth patterns have differential prognoses (Fernández Moro et al., 2018).

### Progression in space and time

Although they are immensely informative, proteomic studies of metastasis-associated ECM have limitations and could benefit from new technical developments. Importantly, the existing approaches do not retain spatial and temporal information – the when and where of ECM dynamics. As shown for primary tumour progression and liver fibrosis, matrisome signatures develop as the cellular composition and relevant signalling pathways change (Baiocchini et al., 2016; Tian et al., 2019). There are technical limitations in isolating smaller early metastases, which are otherwise outweighed by the bulk of normal liver tissue in dECM preparations. Techniques such as laser capture microdissection in combination with mass spectrometry have been used to isolate and profile micrometastases and ECM, and could be useful (Herrera et al., 2020; Elbouchtaoui et al., 2018). This technique can also be used to profile different parts of the tumour, such as the invasive front, to include spatial information (Carnielli et al., 2018).

Multiplexed imaging-based techniques provide an alternative approach. Aiello et al. (2016) used a fluorescent reporter KPC mouse line to characterise the cellular and molecular composition of spontaneous liver metastases of different sizes. They showed that the density of myofibroblasts in metastases increased linearly with metastasis size, and that this correlated with levels of collagen I and the glycoprotein osteonectin. Interestingly, fibronectin and hyaluronic acid density peaked in micro- or milli-sized metastases, challenging the assumptions of runaway fibrosis, even in desmoplasmic metastases such as those of PDAC.

Although definitive studies of the human liver metastasis microenvironment are lacking and would provide a very important perspective on this issue, these existing studies have begun to shed light on early ECM dynamics in the metastatic liver and cellular composition. A holistic understanding of this process will require integration of quantitative ‘omics’ approaches with spatial information across the chronology of metastasis. Additional considerations for matrix proteins studies include how ECM components integrate into tissue architecture and which cells they associate with.

## ECM dynamics in the metastatic cascade

The previous section summarised the known changes of the liver ECM as metastasis progresses, and how some of these changes drive recruitment of different cellular components of the metastatic microenvironment. Here, we outline the current understanding of how these cell-ECM interactions influence the fate of liver metastases.

### Circulating tumour cells and extravasation

To enter the bloodstream, cancer cells must migrate out of the primary tumour site and into the vasculature (intravasation) or at least be able to enter the leaky tumour vasculature. Although this is a critical step in haematogenous metastasis and involves interactions between cancer cells and the BM, it is not thought to be specific to liver tropism and has been reviewed elsewhere (Chiang et al., 2016; Kai et al., 2019). DTCs residing in the blood or lymph are referred to as circulating tumour cells (CTCs), and face a hostile and novel environment to survive in prior to reaching the liver (Blomberg et al., 2018). Indeed, it is estimated that less than 0.02% of DTCs seed metastases, with the rest cleared from circulation either by immune capture, oxidative damage or anoikis, a programmed death due to lack of cell adhesion (Celià-Terrassa and Kang, 2016).

Interestingly, there is emerging evidence that cancer cells with high levels of ECM production can more effectively combat these barriers, as has been shown *in vitro* for drug resistance (Is¸eri et al., 2010). Single-cell RNAseq of CTCs from pancreatic, breast and prostate cancer patients has shown an increase in expression of core matrisome components, including *SPARC*, *MGP* and *SPON2*, that correlated with a shift towards a mesenchymal cell state (Ting et al., 2014). One potential cell survival benefit of enhanced ECM secretion is that it promotes clustering of CTCs with each other and with blood cells to escape anoikis. Indeed, CTC and CTC-platelet clusters have enhanced survival in circulation, and are supported by CTC-derived collagen I (Aceto et al., 2014; Xiong et al., 2020). Clustering can also be supported by cancer-associated fibroblasts through inducing the production of pro-clustering proteins such as the CEACAM proteins (Matsumura et al., 2019). CTC clusters have been isolated from patients and cultured *in vitro* in low-adhesive conditions, although the matrisome signatures from these cultures were not reported (Yu et al., 2014; Cayrefourcq et al., 2015).

Once in the liver vasculature, CTC-derived ECM proteins support extravasation into sinusoids. A recent study investigated the function of two candidate cancer cell-derived matrisome genes, *SERPINB5* and *CSTB*, that were highly expressed in PDAC and associated with poor survival (Tian et al., 2020). Orthotopic injection of a human breast cancer cell line modified for stable knockdown of *SERPINB5* and *CSTB* showed that these two proteins promote liver metastasis owing to enhanced invadopodia formation supporting extravasation. Binding of CTCs to the fibronectin- and collagen IV-rich sinusoid ECM is also a critical requirement for extravasation. In breast cancer liver metastasis, claudin-2 is overexpressed specifically in the liver and enhances binding to sinusoidal ECM through upregulation of α2β1 and α5β1 integrin complexes (Tabariès et al., 2011). Indeed, integrin-mediated binding of DTCs to sinusoidal ECM is important for successful formation of liver metastases (Enns et al., 2004). Thus, both DTC- and liver sinusoid cell-secreted ECM proteins are important in seeding of liver metastases. Whether increased ECM secretion by DTCs is driven by selection or specific intracellular pathways, e.g. mechanosensitive transcription networks, is as yet unclear.

### EMT, growth and migration

Once DTCs arrive in the liver, interactions with the surrounding microenvironment support survival and proliferation. Epithelial-to-mesenchymal transition (EMT; Box 1) describes an orchestrated cell state change that occurs during normal tissue development but can be reinitiated in certain pathological conditions, particularly cancer. Indeed, plasticity along the EMT axis has emerged as a master regulator of many core features of cancer cell behaviour, migration, proliferation and immune escape as metastatic nodules develop (Bakir et al., 2020). Transcriptional changes associated with EMT can be driven by a number of different mechanisms, including transcription factor activation, epigenetic reprogramming and altered proteasome activity. Cell-ECM signalling is a crucial upstream component of these pathways. Stiff environments induce nuclear translocation of YAP/TAZ to activate canonical EMT transcription factors (Zanconato et al., 2019), and promoter methylation of genes that control cell identity, such as *Sox2* (Tan et al., 2014).

Engagement of specific ECM ligand-receptor pathways also promotes EMT in cancer cells. In particular, ECM-integrin pairs such as fibronectin-αVβ3 (Knowles et al., 2013) and tenascin-C-αVβ1/6 (Katoh et al., 2013) drive EMT in breast cancer cell lines. Whether these interactions support EMT of DTCs in liver metastasis has not been clearly elucidated, although fibronectin and tenascin-C are upregulated in metastatic livers (see Table 2). Other ECM proteins enriched in metastatic liver, such as thrombospondin 1, promote EMT in cancer cells to drive migration and invasion, although here the importance of integrin binding is unknown (Liu et al., 2020). Thus, the composition and physical properties of the ECM have a profound impact on cancer cell identity. Whether EMT and invasive behaviour promotes liver metastatic potential generally, or has particular roles in disease progression, requires further study.

**Table 2.**
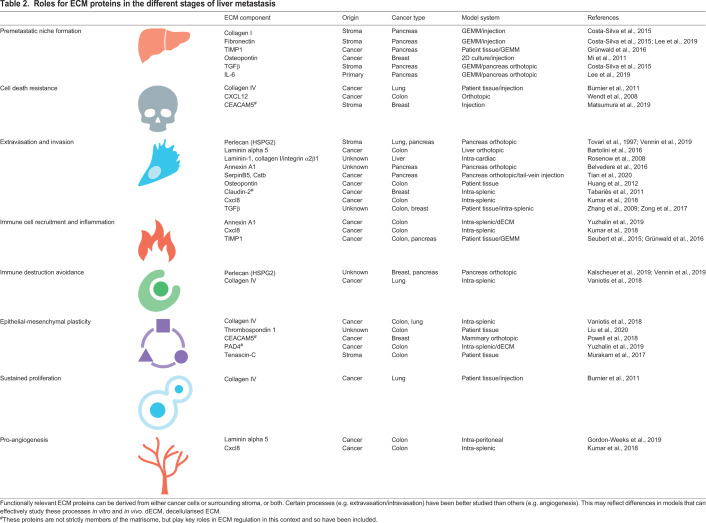
**Roles for ECM proteins in the different stages of liver metastasis**

Preparations of dECM from liver metastases provide a valuable tool to study the direct effects of both physical and ligand-receptor signalling properties of the matrix on cancer cell behaviour. The structure and composition of matrix is highly organ specific, as recently highlighted through dECM analyses of liver and lung tissue (Tian et al., 2018). Seeding CRC cells onto these scaffolds formed metastases that are histopathologically and transcriptionally similar to the *in vivo* CRC metastases that form in these organs. Interestingly, CRC cells cultured on liver ECM scaffolds show a greater propensity to form liver metastases when transplanted *in vivo*, suggesting that the liver matrix either selects or influences liver-tropic cancer cells. D'Angelo et al. (2020) used patient-matched dECM scaffolds from metastatic or healthy liver tissue combined with a CRC cell line to show that the metastatic dECM increased migration and mesenchymal identity of cancer cells compared to the healthy tissue.

A recent study of ECM proteomics from CRC liver metastases showed dramatically increased citrullination of core matrisome proteins, including collagens I, II, III, IV and VI, alongside fibronectin and fibrinogens (Yuzhalin et al., 2018). This enhanced citrullination was due to secretion of the enzyme PAD4 by CRC cells. Interestingly, citrullination of collagen I promotes adhesion and induces epithelial marker expression in CRC cells, suggesting that modifications of ECM proteins play a role in mesenchymal-to-epithelial transition – shown to be important for establishing metastases in the liver (Aiello et al., 2016) and lung (Del Pozo Martin et al., 2015). This illustrates how the metastatic ECM emerges from the interplay between ECM secretion and subsequent modification by both stromal and cancer cells to alter the behaviour of these cell populations.

Although they are useful for identifying cell-ECM effects, dECM preparations lose the interactions between stromal and cancer cells that influence ECM binding, and may release more loosely bound matrisome components. In mitigating the shortcomings of dECM systems, 3D cell culture models have been extremely informative. Gaggioli et al. (2007) grew cancer cell monolayers on a commercial ECM preparation (Matrigel), collagen I and fibroblasts. The fibroblasts led collective invasion of the cancer cells along self-generated tracks of fibrillar collagen and fibronectin (Gaggioli et al., 2007), and have been shown elsewhere to distort the existing BM to facilitate cancer cell migration (Glentis et al., 2017). Indeed, the coupling of activated fibroblasts and cancer cells supports metastatic seeding in the liver and can be therapeutically targeted via inhibition of prolyl hydroxylase domain-containing proteins (Madsen et al., 2015). Spheroid invasion assays are a popular 3D culture setup that enable live imaging of a spheroid containing one or more cell types as they invade into a surrounding matrix-containing substrate. These have been used to live image fibroblast-cancer cell interactions and unpick the specific roles of ECM (Attieh et al., 2017; Miyazaki et al., 2019). One such study showed that αVβ3 integrin binding to fibronectin, rather than the degradation or contractile functions of cancer-associated fibroblasts, was critical to support cancer cell invasion (Attieh et al., 2017). Activated HSCs in the space of Disse may well support invasion of DTCs from the inner sinusoids and into the liver parenchyma in a similar manner.

### Cancer cell metabolism

As well as plasticity of cell identity, cancer cells must also reprogramme their metabolism to adapt to novel sources of energy and provide sufficient ATP production to sustain growth and invasion. Metabolic profiling of metastases formed by the murine breast cancer cell line 4T1 showed that cancer cells forming liver metastases in mice downregulated oxidative phosphorylation programmes in favour of glycolysis, whereas the opposite was true for those forming metastases in the lung (Dupuy et al., 2015).

Cancer cells in the liver utilise abundant extracellular creatine to fuel metastasis (Loo et al., 2015). Interestingly, levels of creatine kinase B in PDAC cells were recently shown to be upregulated in stiff environments and important for the establishment of liver metastases (Papalazarou et al., 2020). This points to an interplay between the increasingly stiff and fibrotic properties of the liver metastatic niche and the metabolic strategies adopted by metastatic cancer cells that seed in the liver. Indeed, many studies have indicated a selection bias for cells adapted for low-oxygen states in the liver, conditions generated by dense ECM and associated with ECM production (Gilkes et al., 2014; Schild et al., 2018).

As well as influencing metabolic state, there is some evidence that ECM proteins can be used by cancer cells as an independent source of energy and nutrients. The amino acid proline is both enriched in many fibrillar ECM proteins and can be a limiting factor in protein translation in cancer cells (D'Aniello et al., 2020). Cancer cells can thus use this exogenous source of proline through ECM degradation and proline uptake pathways, and disrupting this pathway impairs metastasis in breast cancer models (Elia et al., 2017). The links between ECM dynamics and cancer cell metabolism in liver metastases are still poorly understood but remain a fascinating avenue of research and therapeutics.

### Dormancy

DTCs that arrive at metastatic sites can lie dormant for many years in humans before re-emerging (Riggio et al., 2021). These cells are often non-cycling and therefore evade many traditional strategies for targeting tumours, like cytostatic chemotherapy, although there is also evidence for a key role for the microenvironment in fostering the protected nature of dormant DTCs (Ingangi et al., 2019). The dispersed and extremely rare nature of these cells *in vivo* means that progress into understanding dormancy and its niche-specific requirements is particularly challenging.

In the past decade, *ex vivo* microphysiological systems (MPSs) have begun to be used to model the 3D environment, matrix and cellular composition, and flow of nutrients/gases of the metastatic niche. Clark et al. (2014) have made considerable steps towards developing an *ex vivo* MPS of the liver (LiverChip) using primary human hepatocytes and non-parenchymal liver cells as a basis to study hepatic metastatic dormancy. When the highly aggressive breast cancer cell line MDA-MB-231 is introduced into the liver MPS, treatment with the chemotherapeutic agent doxycycline eliminates the majority of cancer cells, leaving a subpopulation of dormant cells. An update to liver MPS introduced a softer, hydrogel-based tissue interface containing canonical fibronectin-binding motifs (Clark et al., 2017) that markedly increased the proportion of dormant cells. This highlights the importance of both mechanical and biochemical cues of the ECM to cancer cell quiescence.

Currently, there are major barriers to wider application of these systems. These include the limited ability to combine MPS models with functional end points such as transcriptomics, proteomics and live imaging. Adapting the structural and compositional complexity of extracellular environments of *in vivo* ECM into MPSs is another challenge. Technologies based on decellularised tissue preparations have begun to close this gap (Ferreira et al., 2020), and simpler, more controlled environments still provide value in addressing specific biological questions.

The role of the ECM in liver metastatic dormancy is still largely unclear. Established *in vivo* models for cancer cell dormancy in the lung suggest that reduced matrix stiffness can promote dormancy, whereas activation of myofibroblasts and increased rigidity promotes emergence (Barkan et al., 2010). Supporting this, HSCs can induce dormancy of PDAC DTCs in the mouse liver via IL-8 signalling, which can be reversed by the transdifferentiation of these HSCs into myofibroblasts (Lenk et al., 2018). Ma et al. used an intrasplenic prostate cancer cell injection model to show that hepatocytes drive mesenchymal-to-epithelial transition in cancer cells, characterised by increased E-cadherin and ERK and by reduced p38, which leads to resistance to chemotherapy and increased liver metastasis (Ma et al., 2016; Ma and Wells, 2014). Analyses of lung metastases have identified roles for fibronectin and collagen I in inducing and awakening dormant cancer cells, which will be important to investigate in the liver (Kai et al., 2019; Barney et al., 2020; Barkan et al., 2010). Taken together, the role of the ECM in dormancy looks set to be a field in which technological advances could play a crucial role in addressing outstanding clinical questions of therapy resistance and disease recurrence.

## Conclusions and future directions

Our understanding of the importance of ECM dynamics in liver metastasis is entering an exciting phase. The topic lies at the intersection between two fields – molecular mechanisms of metastasis and ECM dynamics – that, over the past two decades, have benefited from technical innovations that have dramatically changed the way we think about these processes.

Referring back to the questions we initially posed on the cellular origin, chronology and molecular functions of ECM dynamics in liver metastases, it is clear that significant advances have been made. Proteomic studies over the past few years have helped move our understanding beyond fibrillar-accumulation models and illustrate the plethora of ECM changes that occur at metastatic sites. The use of xenograft models has revealed important contributions of cancer cell-derived ECM, although similar distinctions for different stromal cell populations in the liver are still lacking. Finally, how the ECM microenvironment changes during the early stages of metastasis is not something addressable by current ‘whole-organ’ techniques for ECM proteomics, but would be immensely informative in understanding how pathology progresses.

An emerging question is how best to integrate these ‘omic’ resources with a functional understanding of metastasis. Targeted studies of ECM proteins have illustrated the diverse mechanisms by which matrisome proteins influence metastatic cancer cells and the stroma. Looking forward, *in vitro* systems such as organoids and MPSs that are amenable to high-throughput functionally relevant assays can help identify the ECM proteins important to specific stages of the metastatic cascade, as they have done for drug sensitivity studies (Broutier et al., 2017). Additionally, functional insight from ‘omic’ studies, such as predicting cell-cell interactions from RNAseq data, could help identify key cell-ECM interactions within complex microenvironments *in vivo* (Efremova et al., 2020; Browaeys et al., 2020)*.*

At a microscopic level, the liver can be thought of as a collection of niches that each play a part in metastatic progression. Although good models exist for some of these niches, e.g. extravasation through sinusoidal membranes, there remains scope for progress. How the liver architecture determines the location and development of metastases could be particularly informative here. For example, whether polarisation of stromal cells and ECM along the centro-portal axis affects cancer cell behaviour at different sites within the liver sinusoids is currently unknown. Further, premetastatic changes to the liver may disrupt sinusoid polarity, as has been shown to occur in liver fibrosis (Gissen and Arias, 2015). Application of multiplexed imaging of ECM and cell markers during the early stages of metastasis in experimental models can help provide *in vivo* support for the heterogeneity identified in unbiased screens (Dobie et al., 2019).

Finally, there remains an unmet need for therapies specifically targeting metastatic progression in order to address the main cause of cancer-related mortality. Targeting the ECM microenvironment is an attractive option given the broad influence it has on metastatic progression. Although our understanding is incomplete, an emerging conclusion from existing studies is that the key signalling nodes related to ECM dynamics can dramatically alter the course of metastasis in the liver. TGF-β (Costa-Silva et al., 2015) and IL-6 (Lee et al., 2019) are key signalling molecules in the premetastatic liver niche, and have shown promise in preventing metastasis in preclinical models (Yang et al., 2002). Unfortunately, early clinical trials with TGF-β inhibitors have not been able to recapitulate the preclinical effect sizes, highlighting both the complexity of human disease and the diversity of TGF-β signalling (Teixeira et al., 2020). Other therapies targeting fibrosis in established metastases could also have real clinical importance (Hu et al., 2019), although their differential effects on primary tumours need to be taken into account (Rhim et al., 2014). Ideally, therapeutic targets should specifically disrupt the ECM dynamics that support metastases without disrupting tissue homeostasis. The recognition of ECM contributions from different cell types, particularly cancer cells themselves, offers a promising new approach here. These efforts will be able to integrate the diverse methodologies with which to study the role of ECM dynamics in liver metastasis that are now becoming available.
